# Correction: Correction: The evaluation of a web-based tool for measuring the uncorrected visual acuity and refractive error in keratoconus eyes: A method comparison study

**DOI:** 10.1371/journal.pone.0267585

**Published:** 2022-04-19

**Authors:** The PLOS ONE Staff

There is an error in the Correction published on December 9, 2021. The incorrect figures were published again by mistake in the original Notice of Correction. The publisher apologizes for the error. Please see the complete, correct Figs 1–3 and their captions here.

**Fig 1:**
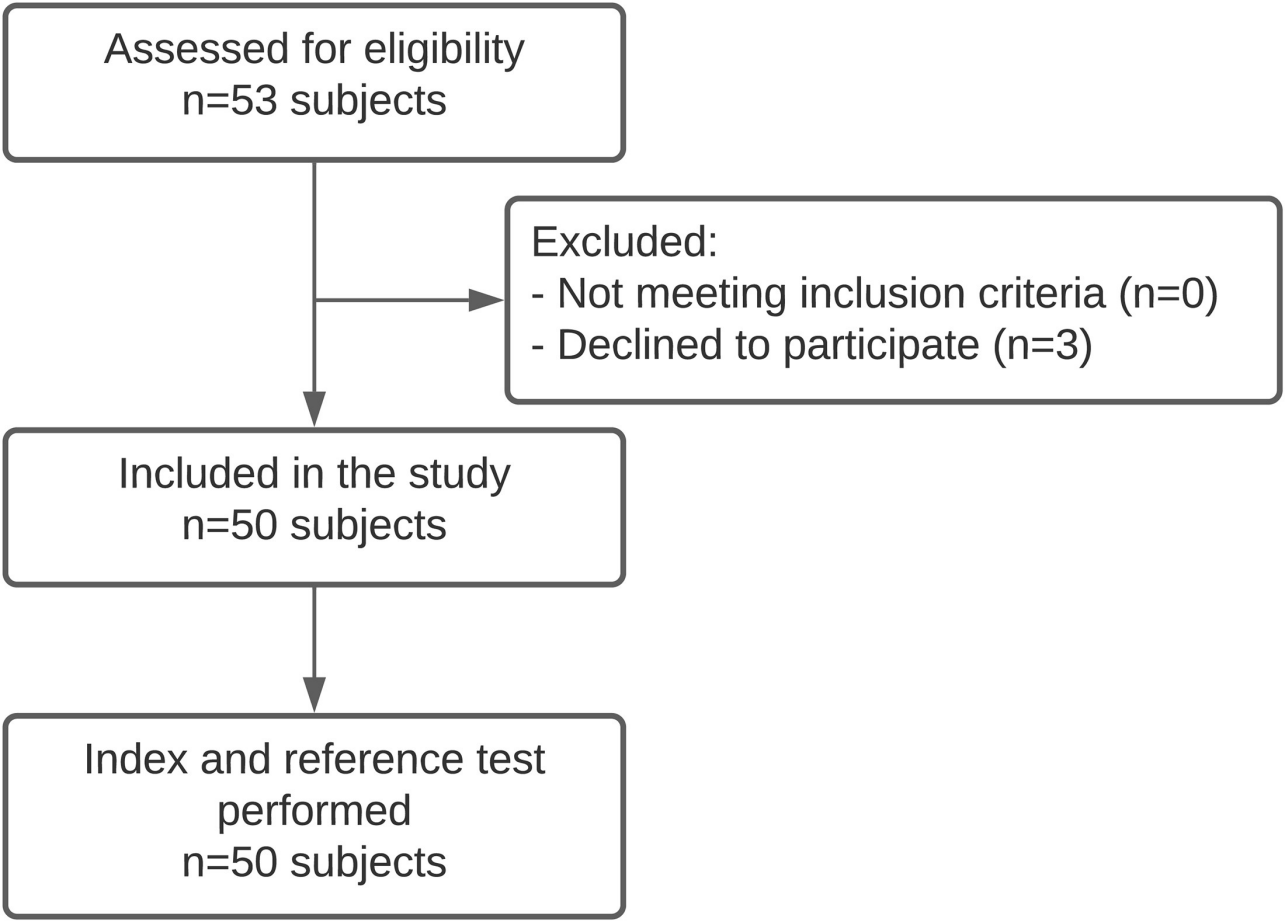
STARD flow diagram illustrating participant flow of the keratoconus population of the MORE-trial. All included participants underwent the web-based (index test) and manifest assessments (reference test) of visual acuity and refractive error.

**Fig 2:**
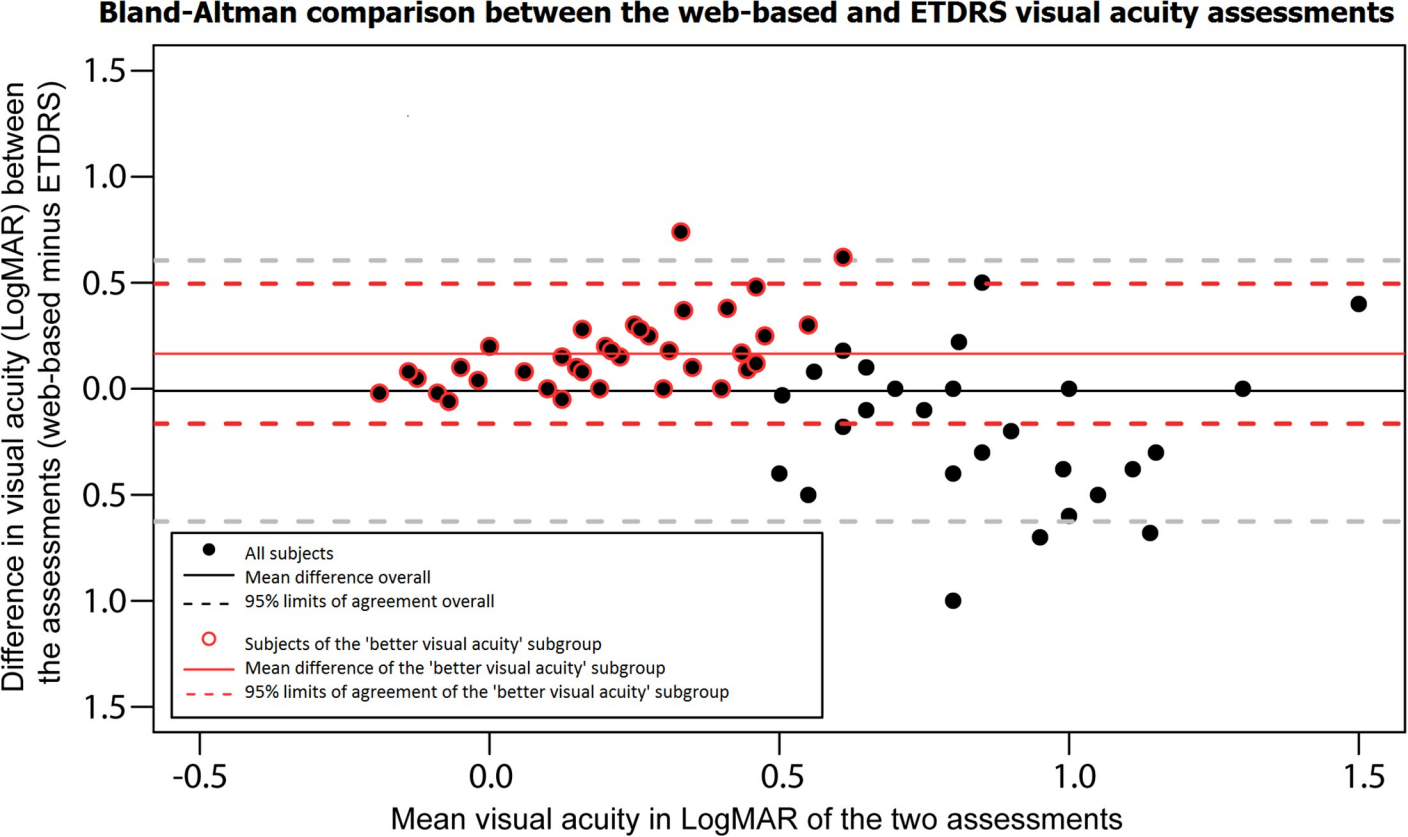
A Bland-Altman plot displaying the differences in logarithmic minimum angle of resolution (LogMAR) between the web-based uncorrected distance visual acuity assessment (index test) and the ETDRS uncorrected distance visual acuity measurement (reference test). The differences between the reference test and index test shown on the Y-axis are expressed as the difference of the web-based uncorrected distance visual acuity assessment outcome minus the ETDRS uncorrected distance visual acuity outcome. The x-axis shows the mean visual acuity in LogMAR of the two assessments, where a more negative value represents a higher visual acuity. The outcome is stratified for a ‘Higher visual acuity’ subgroup (uncorrected distance visual acuity ≤0.5 LogMAR) highlighted with a red circle.

**Fig 3:**
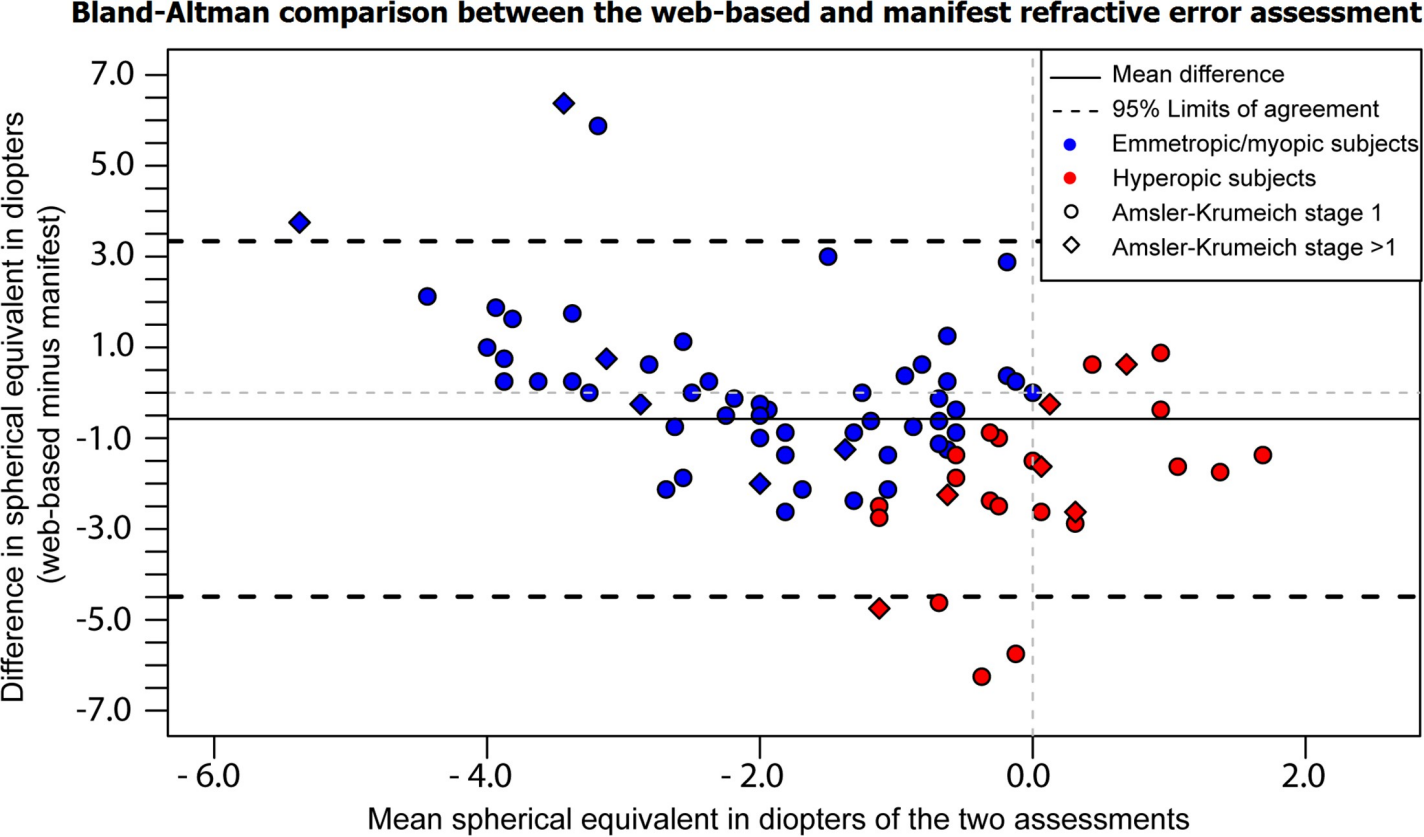
A Bland-Altman plot displaying the differences in refractive error between the web-based refractive assessment (index test) and the manifest refraction (reference test). The difference between the reference and index test shown on the Y-axis is expressed as the difference of the web-based refractive assessment outcome compared to the manifest refraction. The x-axis shows the mean spherical equivalent of the two assessments. Myopia and hyperopia were based on the spherical equivalent of the manifest refraction.
