# Supplementary material for: The evaluation of a web-based tool for measuring the uncorrected visual acuity and refractive error in keratoconus eyes: A method comparison study
Source: PLoS One. 2021 Aug 18;16(8):e0256087. doi: 10.1371/journal.pone.0256087 (PMC8372909; doi:10.1371/journal.pone.0256087)
Supplement: Attachment — Submitted filename: Rebuttal.docx [file pone.0256087.s006.docx]

**Manuscript ID: PONE-D-20-36881R1**

**Subject: rebuttal for manuscript “The evaluation of a web-based tool for measuring the uncorrected visual acuity and refractive error in keratoconus eyes: a prospective open-label method comparison study”**

Reviewers' comments:

Reviewer #3: All comments have been addressed

Reviewer #2: All comments have been addressed

Reviewer #1:

Line 36 306: visual acuity measurement is not objective. What is the typical intra-individual variation of the web-based test in this patients? (0.1logMAR as with standard acuity test)? As you discuss this in line 306 for VA measurements in subjects with no ocular diseases, do you have data on subjects with keratoconus?

*Reply:* We believe that a visual acuity assessment will enhance teleconsultations, but agree with the reviewer a visual acuity test is not an objective measurement and thus removed this word from the abstract and manuscript and replaced it with “quantifiable” (as it provides the health care professional with an exact value).

We did not investigate the test-retest performance of the web-based assessment, so the intra-individual variation between measurements is unknown. The reported variation between visual acuity measurements that we refer to in the manuscript is derived from a study performed in an eye clinic in patient with various ocular diseases, however, no data specifically for keratoconus patients is available.

Line 38 and following: statement biased by the sponsor? I doubt that algorithm is trainable with this data.

*Reply:* We thank the author for this comment. It is indeed true that the algorithm’s calculations cannot with this inadequate data, so we agree that this statement needs some nuance. The study has provided us with valuable insights in the limitations of this web-based tool. We will use this knowledge to increase overall safety of the web-based tool by better identification of outlier cases. We have adjusted this sentence in the Abstract Conclusion and Manuscript conclusion accordingly.

General: when you only measure VA, how easy is it to diagnose for keratoconus, especially in early stages? Its not, or? In case of “telemedicine” any drop in VA is most likely related to a refractive error and no diagnosis can be made w/o corneal topography. Is the last section of the introduction still valid having that in mind?

*Reply:* We agree that using visual acuity only keratoconus cannot be diagnosed and to our knowledge we do not claim or suggest this in the manuscript. The aim and focus of this study was to evaluate the performance of the algorithm in complex refraction. Keratoconus patients often/always have a complex refraction (e.g., irregular astigmatism) and were therefore a suitable study population for our research question. In the general population, complex refractive errors are also present with or without an underlying eye condition and this study provided insight in the limitations and accuracy (of in this case inaccuracy) of the algorithm in these cases.

Line 284: please change the order and put the limitation on the number of available measurements first. This makes it easier to the reader to classify the results. Please also put the number of cases in brackets for available measurements.

*Reply:* We thank the reviewer for this comment. We have changed the order of the discussed limitations as suggested by the reviewer. We have added the “13/100” in between brackets to clarify the ratio between missing data and available measurements.

Line 318, please add: as such with keratoconus of different stages.

*Reply:* We’ve revised the sentence and included this suggestion.

Line 361: is this statement true for patients without diseases? Please be specific

*Reply:* We revised the sentence to make it clear we are referring to our study outcomes. In addition, to answer the question; it is indeed true that visual acuity can be more reliably assessed in better visual acuity ranges regardless of ocular conditions.

Line 364: results did not only resulted in poor visual performance (as in terms of VA), but also in a correction that would not be acceptable (in terms of diopters).

*Reply:* For clarification, we have included in the conclusion that the web-based refractive assessment was found to be inferior to the manifest assessment. However, we deem a prescription unacceptable based on visual performance rather than the exact dioptric values as these can differ without directly affecting visual performance.

Line 368: Again: VA is subjective, not objective.

*Reply:* see comment 1.

Other changes:

We replaced “higher visual acuity” subgroup with “better visual acuity” in the legend and footnote of figure 2, so it is now similar to the manuscript.
